# The relevance of tyrosine kinase inhibitors for global metabolic pathways in cancer

**DOI:** 10.1186/s12943-018-0798-9

**Published:** 2018-02-19

**Authors:** Michaela Poliaková, Daniel M. Aebersold, Yitzhak Zimmer, Michaela Medová

**Affiliations:** 1 Department of Radiation Oncology, Inselspital, Bern University Hospital, and University of Bern, Bern, Switzerland; 2 Department for BioMedical Research, Inselspital, Bern University Hospital, and University of Bern, Bern, Switzerland

**Keywords:** Tyrosine kinase inhibitors, Metabolomics, Targeted therapies, Glycolysis, Glucose, TCA cycle, Energy metabolism, Amino acids, Lipid metabolism

## Abstract

Tumor metabolism is a thrilling discipline that focuses on mechanisms used by cancer cells to earn crucial building blocks and energy to preserve growth and overcome resistance to various treatment modalities. At the same time, therapies directed specifically against aberrant signalling pathways driven by protein tyrosine kinases (TKs) involved in proliferation, metastasis and growth count for several years to promising anti-cancer approaches. In this respect, small molecule inhibitors are the most widely used clinically relevant means for targeted therapy, with a rising number of approvals for TKs inhibitors. In this review, we discuss recent observations related to TKs-associated metabolism and to metabolic feedback that is initialized as cellular response to particular TK-targeted therapies. These observations provide collective evidence that therapeutic responses are primarily linked to such pathways as regulation of lipid and amino acid metabolism, TCA cycle and glycolysis, advocating therefore the development of further effective targeted therapies against a broader spectrum of TKs to treat patients whose tumors display deregulated signalling driven by these proteins.

## Background

The switch from normal tissue to malignancy is a result of oncogenes-driven biochemical processes aimed at sustaining an accelerated rate of proliferation and growth [1]. Otto Warburg in 1956 described for the first time a specific metabolic characteristic of neoplasms by demonstrating that a cancer cell, unlike an untransformed cell, relies mainly on a higher glycolytic flux without a change in oxidative phosphorylation even in the presence of oxygen [2]. The so-called Warburg effect is nowadays considered a major hallmark of cancer and numerous studies have been repeatedly reporting that various metabolic pathways appear to be distinctive in individual tumour cells [3, 4]. Many of these alterations emerge as a consequence of the gain of mutations accumulated during oncogenesis, providing proliferative advantage for cancer cells in their microenvironment.

In recent years, in addition to investigating the role of cell metabolism in tumor cell development, particular attention has been devoted to metabolic changes occurring as a response to targeted treatments [5–7]. In view of the role that TKs seem to play in the regulation of cellular metabolism [8–11], it is crucial to determine whether the antitumor activity of particular tyrosine kinase inhibitors (TKIs) is related to their effect at a given metabolic level. Such insights may subsequently serve as an important ground for novel personalized therapeutic options and combination treatments. Assessment of biological conformity in changes in metabolites following administration of a particular TKI has already shown to provide important translational observations as to particular sensitive metabolic pathways [12]. Consequently, metabolomics has the potential to identify subgroups of patients that are likely to profit from given targeted perturbations and, of a similar importance, determine subgroups that may encounter toxicity or resistance.

Protein kinases constitute an immense enzyme family that emerges as a strikingly valuable set of targets in therapy of various tumors considering their high sensitivity to specific kinase inhibitors, which are often relatively well tolerated by normal cells. Development of TKIs created a therapeutic window for selective diminishing of malignancies with constitutively active kinase. The majority of these compounds share a common mechanism of action – they competitively inhibit adenosine triphosphate (ATP) at the catalytic binding site of the targeted protein [13]. As aforementioned, accumulating evidence suggests that key oncogenic pathways program the adaptation of metabolism with explicit changes for the selective advantage of tumor cells, many of them regulated by tyrosine kinase activity [14–16]. In this review, we summarize and discuss principal metabolic changes following administration of particular kinase inhibitors on different levels of cellular metabolism (key metabolites and molecules affected by TKIs in cancer are summarized in Table 1).

**Table 1:** Summary of key metabolites and molecules affected by TKIs in cancer. Up- or downregulation highly depends on the inhibitor and model of the study used

Metabolite	Function in	Sense of Regulation	Rerence(s)
Fructose 1,6-bisphosphate	glycolysis	**↓**	[27, 34, 38, 41, 45, 48, 49, 58]
Dihydroxyacetone phosphate			
3-phosphoglycerate			
Glucose (consumption)			
Phosphoenolpyruvate	glycolysis and gluconeogenesis	**↓**	[22, 27, 38, 45]
Lactate			
Glyceraldehyde 3-phosphate			
Pyruvate			
6-phosphogluconate	pentose phosphate pathway	**↓**	[27, 58]
Ribulose-5-phosphate			
Ribose-5-phosphate			
Xylulose-5-phosphate			
D-sedoheptulose 1,7-bisphosphate	pentose phosphate pathway	**↑**	[34]
Deoxyribose phosphate			
Glucose-6-phosphate	glycolysis and PPP	**↓**	[27, 58]
Glutamate	amino acid metabolism	**↑**	[27, 30, 34, 45, 74]
Valine			
Lysine			
Tyrosine			
Aspartate			
Proline			
Threonine			
Histidine			
Asparagine			
Tryptophan			
Alanine			
NADPH	pentose poshosphate pathway	**↓**	[34, 51]
	oxidation-reduction pathways		
ATP, GTP, CTP, TTP	energy metabolism	**↑**	[32, 45, 58]
Fumarate	TCA cycle	**↓**	[27, 30]
Malate			
Citrate			
Arginine	amino acid metabolism	**↓**	[74, 76]
Citrate	TCA cycle	**↑**	[32]
ATP	energy metabolism	**↓**	[60, 62]
Phosphocholine	glycerophospholipid metabolism	**↓**	[45, 87–89]

Abbreviations: ***↑****—*Up-regulation; **↓***—*Down-regulation; *TCA cycle* Tricarboxylic acid cycle; *NADPH* Nicotinamide adenine dinucleotide phosphate; *ATP* Adenosine triphosphate; *GTP* Guanosine triphosphate; *CTP* Cytidine triphosphate; *TTP* Thymidine triphosphate

## Impact of TKIs on Glycolysis and glucose-related pathways

As metabolic reprogramming towards aerobic glycolysis has been suggested as one of the hallmarks of cancer, considerable research efforts focused for over a decade on enzymes and metabolites of the glycolytic pathway following antineoplastic treatments. Glucose metabolism, a paramount energetic resource for the cell, is a very complex process regulated in neoplastic cells by different oncogenes on multiple levels, ranging from transcription to post-translation modifications [14]. In that respect, for example, c-MYC controls key metabolic enzymes including those that are involved in glucose metabolism such as hexokinase 2 (HK2), glucose transporter 1 (GLUT1), pyruvate kinase muscle isozyme 2 (PKM2) and lactate dehydrogenase A (LDHA) [17].

**Fig. 1:**
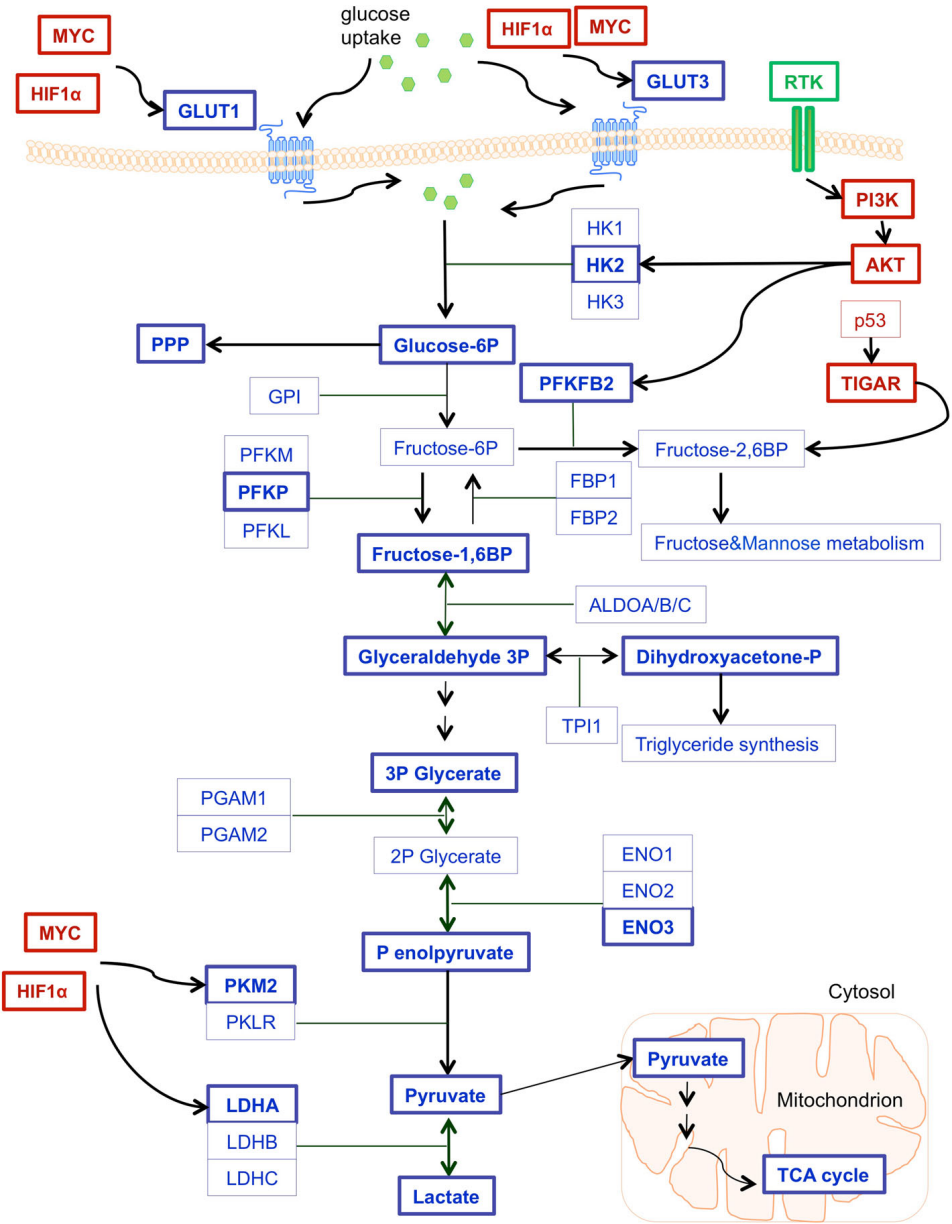
TKI-induced regulation of glycolytic pathway. Highlighted in bold are proteins and metabolites (blue) together with glycolytic regulators (red) that were shown to be affected by the inhibition of TKs. Abbreviations: GLUT1/3*—*glucose transporter 1/3; HK1/2/3*—*hexokinase 1/2/3; TIGAR*—*TP53-inducible glycolysis and apoptosis regulator; P*—*phosphate; BP*—*bisphosphate; PPP*—*pentose phosphate pathway; GPI*—*glucose-6-phosphate isomerase; PFKFB2*—* 6-phosphofructo-2-kinase/fructose-2,6-bisphosphatase 2; PFK*—*6- phosphofructokinase(three isoforms – muscle (PFKM), liver (PFKL) and platelet (PFKP)); FBP1/2*—*fructose-bisphosphatase 1/2; ALDOA/B/C*—*aldolase A/B/C; TPI1—triosephosphate isomerase; PGAM1/2—phosphoglycerate mutase 1/2; ENO1/2/3—enolase 1/2/3; PKM2—pyruvate kinase isozyme M2; PKLR—Pyruvate kinase isozymes L/R; LDHA/B/C—lactate dehydrogenase A/B/C; TCA cycle*—*tricarboxylic acid cycle

Oncogene-conducted activation of glycolytic pathway takes frequently place through hypoxia-inducible factor 1α (HIF-1α) [18, 19]. The already mentioned Warburg effect is a result of deregulated genes, leading to upregulation of glucose transporters 1 and 3, with resulting elevated glucose consumption [20, 21]. Glucose metabolism does not necessarily encompass glycolysis only. Indeed, other glucose-related metabolic pathways, as the pentose phosphate pathway (PPP), which provides nicotinamide adenine dinucleotide phosphate (NADPH), the hexosamine pathway, a minor branch of glycolysis needed for glycosylation of proteins, and glycogenesis that generates glycogen used as a glucose repository, are all critical branches of cellular glucose metabolism [22]. Since it has been shown that many RTKs inhibitors suppress among others also metabolic pathways as for example the PI3K/Akt pathway, it is expected that they would inhibit glucose metabolism in a similar manner [23, 24]. In this section we summarize how glycolysis and other glucose-related pathways are reprogrammed in malignant cells following particular TKI targeting (summarized in Fig. 1).

### ErbB family

#### Epidermal growth factor receptor (EGFR)

EGFR, a broadly studied RTK system, is overexpressed, deregulated and mutated in a large number of malignancies. Specifically, EGFR protein overexpression was detected in tumors of breast, brain, cervix, ovary, colon, head and neck and lung [25, 26], creating a strong motivation to develop novel antitumor agents focused on EGFR.

The 2014 study from Makinoshima and collaborators [27] provided one of the first comprehensive analyses of EGFR TKI-mediated modulations of metabolism. The presence of EGFR TKIs erlotinib (Tarceva®) and gefitinib (Iressa®) repressed lactate production and glucose consumption in three distinct lung adenocarcinoma (LAD) cell lines, HCC827, NCI-H1975 and PC-9 [27]. Importantly, HCC827 and PC-9 both carry the EGFR exon 19 delE746-A750 mutation and are sensitive to EGFR TKIs whereas H1975 harbors the EGFR L858R + T790 M mutation, which causes resistance to both gefitinib and erlotinib [28]. The authors hypothesized that lactate production is regulated by MYC via transcriptional regulation, since MYC is decreased at both protein and mRNA levels following treatment by EGFR TKIs. Interestingly, western blot analysis showed that MYC-regulated proteins HK2 and GLUT3, but not GLUT1, were reduced in EGFR TKI-sensitive cell lines upon treatment [27]. Metabolome analysis using Capillary Electrophoresis Time of Flight Mass Spectrometer (CE-TOFMS) exposed intermediate key metabolites in glucose metabolism that were altered following erlotinib treatment in both EGFR TKI-sensitive cell lines HCC827 and PC-9. Specifically, fructose 1,6-bisphosphate (FBP), dihydroxyacetone phosphate (DHAP), 3-phosphoglycerate (3PG), phosphoenolpyruvate (PEP), lactate (LA), and 6-phosphogluconate (6PG) were all decreased in TKI-sensitive HCC827 and PC9 cells after 6 h of erlotinib treatment, but not in TKI-resistant NCI-H1975 cells [27]. Furthermore, PPP metabolites, glucose 6-phosphate (G6P), glyceraldehyde 3-phosphate (G3P), pyruvate (PA), ribulose 5-phosphate (Ribu5P), and ribose 5-phosphate (R5P) were significantly reduced in both HCC827 and PC9 cells [27]. Measuring the extracellular acidification rate (ECAR), an indirect readout of the glycolytic rate, Lim et al. reported an attenuation of ECAR by the co-treatment with EGF stimulation together with gefitinib in an EGFR-overexpressing breast cancer cell line MDA-MB-468 [29]. Moreover, they showed that EGFR binds, phosphorylates and inhibits PKM2, a rate-limiting glycolytic enzyme that catalyses the last glycolysis step [29]. On the contrary, ECAR was increased in triple-negative breast cancer (TNBC) mesenchymal-like cell lines MDA-MB-231 and Hs578T upon treatment by erlotinib or the MET inhibitor capmatinib (INC280) [30]. The impact of EGFRi on glycolysis was further confirmed by the Heath group in 2015, who reported, as assessed by the ^18^F–FDG radioassay, a reduction of consumption of glucose and hexokinase activity following erlotinib treatment in patient-derived glioblastoma (GBM) neurosphere tumor cells (GBM39) that express EGFR [31]. Outlining similarities with other authors’ models, further recent report conducted by De Rosa et al., where one EGFR inhibition-sensitive cell line (HCC827) and two EGFR inhibition-resistant cell lines (H1975 and H1993 (both bearing MET gene amplification)) were exposed to WZ4002 (a specific EGFR^T790M^ inhibitor), erlotinib or PHA665752 (a first generation MET inhibitor) and their impacts on glycolytic enzymes and transporters were investigated [32]. Although protein levels of HKI, PKM1/2 and GLUT1 remained consistent across all cell lines, all three studied inhibitors led to a concentration-dependent downregulation of HKII and to upregulation of levels of GLUT3 with efficient inhibitors of the corresponding cell line (curiously, the levels of GLUT3 were upregulated after 72 h treatment of H1975 with WZ4002 or following treatment of H1993 cells with PHA665752) [32]. Moreover, a reduction of pPKM2 was observed in HCC827 and H1993 treated with erlotinib and PHA665752, respectively [32]. The in vitro observations were further substantiated in vivo by using H1975 and H1993 cells injected into female BALB/c (nu/nu) mice treated with WZ4002 and crizotinib (Xalkori® a MET inhibitor), respectively [32]. This differential regulation of glycolysis brings a rationale for a potential combination therapy targeting both the EGFR pathway and glucose metabolism for enhanced therapeutic effect [32]. Interestingly, the impact of EGFR inhibition on glucose-related metabolism was recently substantiated both in cell culture and in vivo using HCC827 and H1650 (bearing E746-A750 deletion of exon 19) cell lines, where erlotinib therapy reduced expression of MYC and HIF1α and their downstream targets GLUT1, HKII, neutral amino acid transporter B(0) (SLC1A5) together with sodium-coupled neutral amino acid transporter 1 (SLC38A1) [33]. These results further correlated with decreased ^18^F–FDG and ^11^C–Gln uptake seen in HCC827 xenografts following the erlotinib treatment [33]. In addition, metabolic profiling of myeloma cancer cells LP-1 (no NRAS, KRAS or BRAF mutation), L-363 (harbouring NRAS mutation), RPMI-8226 (KRAS mutation), and U-266 (BRAF mutation) revealed that following the treatment with gefitinib, metabolites from the PPP such as ribose-phosphate, D-sedoheptulose-1,7-bisphosphate, O8P-O19 and deoxyribose phosphate were significantly increased in LP-1 cell line and unchanged in KRAS/NRAS/BRAF mutant myeloma cancer cells [34]. As PPP is a main source of NADPH supplying R5P for nucleotide synthesis, the authors hypothesized that the upregulation of these metabolites is a metabolic compensatory mechanism to prevent complete therapeutic response towards EGFR inhibition [34]. This hypothesis was experimentally confirmed by the use of the antimetabolite 6AN, a PPP inhibitor, together with gefitinib [34] The combinational therapy supressed the proliferation of LP-1 cells, which was recovered by supplementation of NADPH. Analogous results were reported using afatinib (Giotrif®), a dual EGFR and ERBB2 inhibitor [34] as well as in another study, where MET or EGFR inhibition both sensitized TNBC cell line MDA-MB-468 to nucleotide enzymes knockdown [30].

#### HER2

Similarly to EGFR, HER2, encoded by the *ERBB2* gene, is also often overexpressed in cancer and its deregulation is associated with aggressive phenotype and shortened survival [35]. Targeting HER2 by the humanized murine monoclonal antibody trastuzumab (Herceptin®) leads to a 40% improved overall survival in patients with breast cancer that show approximately 15%–25% amplification or overexpression of HER2 [36, 37].

Zhao et al. reported that trastuzumab inhibits glucose uptake and lactate production in BT474 and ZR-7530 breast cancer cell lines without a change in cell growth inhibition, hypothesising that glycolysis inhibition is not a consequence of the cell growth inhibition [38]. Their previous study showed that the ErbB2-heat shock factor1 (HSF1)-lactate dehydrogenase A (LDHA) pathway has a main role in glucose regulation in breast cancer cells [39]. Therefore they suggested and subsequently also reported that trastuzumab inhibits glycolysis through downregulation of the HSF1-LDHA axis and, moreover, this axis contributes to the resistance of breast cancer cells to this monoclonal antibody [38]. Similar response on glycolysis was shown with lapatinib (Tykerb®), a dual inhibitor of EGFR and ErbB2/HER2 that is usually used in combination with capecitabine for the treatment of HER2-positive metastatic breast cancer [40]. Specifically, Komurov et al. reported that lapatinib treatment of ErbB2-positive SKBR3 breast cancer cells induced glucose deprivation, suggesting a blockage of glucose-dependent EGFR/HER2 signaling [41]. Additional study by Ruprecht et al. unveiled that phosphorylation of Ser466 of 6-phosphofructo-2-kinase/fructose-2,6-bisphosphatase 2 (PFKFB2) is inhibited following lapatinib treatment in lapatinib-sensitive BT-474 breast cancer cell line, however it recovers to its initial levels of phosphorylation in lapatinib-resistant BT-474 clone BT-474-J4 [42]. Phosphorylation of Ser466 was reported to trigger PFKFB2 kinase activity that activates the production of metabolite fructose-2,6-bisphosphate, pointing out a possible link between lapatinib therapeutic action and metabolic reprogramming in resistance [42].

The results of research efforts focusing on ErbB2 family of RTKs strongly suggest that the decrease of intermediate metabolites in PPP and glycolysis such as lactate, FBP, G6P or R5P and the impairment of glycolysis-related enzymes such as GLUT1 and HK1 are not events resulting from inhibited proliferation but could potentially serve as biomarkers to predict the response to and, more importantly, efficacy of EGFR and HER2 TKI treatment.

### BCR-ABL

BCR-ABL harbours a constitutively active form of the ABL TK and is present in more than 90% of chronic myeloid leukemia (CML) patients [43]. CML treatment was revolutionized by the use of the BCR-ABL TKI imatinib (formerly STI571, Gleevec®), a compound that was writing the first success stories in the field of targeted neoplastic treatment [43]. Imatinib provides effective and durable therapy: the treatment resulted in 5-year survival of approximately 90% for CML patients in clinical trials [44].

In 2004, Gottschalk et al. reported that imatinib treatment changed glucose metabolism from anaerobic glycolysis to the aerobic mitochondrial TCA cycle in two human BCR-ABL-positive cell lines CML-T1 and K562 but not in BCR-ABL-negative cell line HC-1 [45]. Interestingly, metabolic responses to imatinib were dependent on the concentration of the molecule. When using a concentration of 0.25 μmol/L, which is below imatinib’s IC_50_ value (for CML-T1 IC_50_ is 0.69 ± 0.06 μmol/L and for K562 IC_50_ is 0.47 ± 0.04 μmol/L), lactate production was reduced in BCR-ABL-positive cell lines and concurrently, the production of glutamate increased, thus suggesting increased employment of the mitochondrial glucose pathway; when using a concentration above its IC_50_ value (2.5 μmol/L), no activation of TCA cycle was observed [45]. Moreover, imatinib was able to increase extracellular glucose in the lyophilized media of BCR-ABL-positive cell lines in contrary to media coming from the BCR-ABL-negative cell line, where the concentration of extracellular glucose did not change [45]. Consequently, this resulted in an increased ratio of extracellular to intracelullar glucose and decreased glucose uptake in BCR-ABL-positive cells [45]. These data correlate with previous findings of Boros et al. who showed that imatinib regulates glycolysis through downregulation of GLUT1 in human leukemia cells [46]. In fact, BCR-ABL-positive haemopoietic cells TonB210 express high affinity GLUT1 and demonstrate increased glucose uptake [47]. Following the treatment in vitro, imatinib led to the internalization of 90% of GLUT1 and drastically decreased hexose uptake [47]. A study carried out by the group of Serkova et al. aimed at understanding of development of imatinib resistance metabolic phenotype in CML, using imatinib-sensitive K562-s and LAMA84-s and imatinib-resistant K562-r and LAMA84-r cell lines [48]. By using nuclear magnetic resonance spectroscopy and gas chromatography mass spectrometry to assess ^13^C glucose uptake and metabolism, they showed that in both imatinib-sensitive cell lines, imatinib treatment (1 μmol/L) significantly decreased glucose uptake and lactate export together with reduced [4-^13^C]glutamate, in contrast to imatinib-resistant cell lines, suggesting a decrease in the activity of glycolysis together with TCA cycle [48]. To confirm their findings, they used 2-deoxy-d-glucose uptake assay and showed that imatinib-sensitive cell lines displayed decreased uptake of glucose, compared to imatinib-resistant cell lines that exhibit even higher glucose uptake, as a possible consequence of imatinib resistance progress [48]. To explain the drop in glucose uptake in imatinib-sensitive cell lines, they reported that imatinib inhibits glycolysis and translocates GLUT1 from the membrane into the cytosol, whereas GLUT1 remains located at the plasma membrane in resistant cell lines [48]. Interestingly, a decrease in 18-fluoro-2-deoxy-D-glucose (FDG) uptake was previously described in a case report of a patient with a jejunal gastrointestinal stromal tumor with multiple hepatic metastases treated with imatinib [49].

Studies employing BCR-ABL targeted therapy provided a rationale for the combined use of an inhibitor of glucose metabolism and kinase inhibitors for treatment of BCR-ABL-positive patients who acquired resistance either to classical chemotherapy or to the targeted treatment.

### Met

The MET RTK for hepatocyte growth factor (HGF) is, analogously to other RTKs, actively involved in cell growth, migration and proliferation and additionally functions also as a main regulator of embryogenesis [50]. In a study published in 2011, Lui et al. used two nasopharyngeal cancer (NPC) cell lines, HK1-LMP1 and CNE-2, and described that protein levels of regulator of apoptosis and glycolysis, TP53-induced Glycolysis and Apoptosis Regulator (TIGAR), were reduced after the treatment with two MET TKIs (by AM7, a MET inhibitor binding to the kinase linker region and extending to a hydrophobic binding site and by a tool compound SU11274), indicating that the effect is induced by METi itself and does not depend on the exact nature of inhibitor used [51]. Previously, it was proposed that TIGAR inhibits apoptosis by regulation of cellular NADPH levels and via regulation of the PPP [52]. Indeed, they explored METi reduction of intracellular NADPH, a protector from oxidative stress and a driver of the force of most biosynthetic enzymatic reactions, responsible for biosynthesis of DNA, RNA, cholesterol and fatty acids [53, 54], in both NPC cell lines [51]. Interestingly, using a METi-sensitive SNU5 and METi-resistant SNU1 gastric cancer cell lines, expression of several glycolysis-related mitochondrial enzymes, such as voltage-dependent anion-selective channel protein 1 (VDAC1) and adenine nucleotide translocase 2 (ANT2), was significantly regulated in response to MET inhibitor PHA665752 [55]. Impact of MET inhibition on glucose metabolism was confirmed using H1975 NSCLC cancer cells in a xenograft model (Ncr-nu mice) monitored in vivo by FDG-PET (glucose analogue [^18^F]fluoro-2-deoxy-D-glucose-positron emission tomography) analysis with MRI [56]. Indeed, MET inhibitor SU11274-treated xenografts displayed a 45% drop in glucose metabolism compared to untreated controls [56].

In conclusion, analogously to findings pertaining to inhibition of ErbB2 family of receptors, MET inhibition also seems to modulate glucose metabolism and this observation could potentially serve as a mean to predict cancer cells responses to MET targeting-based treatments.

### Other protein TKs

Anaplastic lymphoma kinase (ALK) is engaged in the induction and progression of various cancer types, including non-small cell lung cancer (NSCLC), neuroblastomas and lymphomas. ALK is usually targeted in clinical practice by crizotinib, approved for use in ALK-positive NSCLC [57]. Some preliminary work on impacts of ALK inhibition on cellular metabolism was carried out by McDonnell et al., focusing on anaplastic large cell lymphoma (ALCL) cell lines SU-DHL-1, DEL, Karpas299, SUPM2 and using ALK inhibitor CEP-26939 (CEP, unknown mechanism of action, Cephalon) [58]. Metabolomic analysis by both gas chromatography–mass spectrometry and liquid chromatography-mass spectrometry displayed a significant decrease in lactate following 3 h of treatment by 300 nM of CEP, which was accompanied by a decrease in phosphorylated LDH detected by phosphoproteomics via metal oxide affinity chromatography (MOAC) [58]. Using ^13^C–glucose, they could demonstrate that lactate in these cell lines was derived directly from glucose, suggesting reduction of glycolytic flux following ALK inhibition. Moreover, reduced glycolytic flux occurred to be due to a decreased glucose uptake and reduced metabolites such as FBP, G6P and F6P [58]. In addition, ribose-5-phosphate and xylulose-5-phosphate, main metabolites in PPP, were significantly downregulated following inhibition of ALK [58]. On contrary, no similar metabolic changes were detected in ALK-negative Jurkat cells treated by CEP, used as a negative control [58]. Of clinical importance is the fact that comparable results were observed also using crizotinib [58]. Altogether, the data in this study provided a rationale that PKM2 is functioning as a mediator of ALK-regulated metabolic switch as inhibition of ALK resulted in reduction of pY105 PKM2, without a change in total PKM2 levels [58].

Differently from what was reported previously using other TKIs, Hudson and colleagues treated mouse pancreatic ductal adenocarcinoma (PDAC) cell lines from pancreatic cancer mouse model (Kras^G12D^Pdx1-cre) with axitinib (Inlyta®, mechanism of action through VEGFR, c-KIT and PDGFR) and did not observe the expected effect on glycolysis and [C-14] deoxyglucose uptake was increased in axitinib-treated cells after 24 and 48 h [59]. It has to be considered, however, that these experiments were performed with axitinib-resistant PDAC clones, surviving after longer incubation times or higher concentrations of axitinib [59]. These results suggest that the increased glucose uptake following axitinib treatment is involved in the resistance mechanism towards the inhibitor-induced anti-cancer effect. Moreover, the treatment with increasing concentrations of axitinib upregulated GLUT1 together with ECAR, proposing a way through which axitinib induces glucose uptake [59].

Sorafenib (Nexavar®), a multikinase inhibitor targeting BRAF, PDGFR and VEGFR, enhanced in the hepatocholangiocarcinoma cell line LCSC-2 the expression of GLUT3, Enolase 2 (*ENO2*), and the platelet phosphofructokinase (*PFKP*), three genes directly associated with glycolysis, hence suggesting a metabolic shift towards glucose metabolism [60]. Indeed, the response to sorafenib also induced uptake of the fluorescent glucose analogue 6NDBG, glucose consumption and lactate production [60]. The gene signature that emerges following treatment with sorafenib indicates an induction of glycolytic readjustment as a response to mitochondrial collapse [60].

In another study, FGFR1 inhibition by TKI258/dovitinib, a multikinase inhibitor (VEGFR, FGFR, PDGF, c-KIT, CSF-1R), significantly increased enzymatic activity of PKM2 in human myeloid leukemia cell line KG1, breast cancer cell line MDA-MB-134 and a lung cancer cell line NCI-H1299, all three of them overexpressing FGFR1 [61]. Additional data that suggest a role for FGFR1 in modulating glucose energy metabolism were provided recently by Fumarola et al. [62]. Using squamous cell lung cancer (SQCLC) cell lines H1703 and H520 after FGF2 induction, they could show that the protein expression of both HIF-1α and GLUT1 correlated with elevated glucose uptake, glycolysis, lactate production and elevated PKM2 activity. Treatment with a selective FGFR inhibitor NVP-BGJ398 or with a multikinase inhibitor dovitinib hindered all these processes, pointing towards AKT/mTOR pathway as a key player in this regard. Importantly, the involvement of FGFR1 signaling affecting glucose metabolism was equally confirmed in vivo with LENTI-4 cells with FGFR1 amplification generated from SQCLC SKMES-1 cells by lentiviral expression [62].

## TCA cycle and energy metabolism

TCA cycle is commonly presented in a simple viewpoint of a cyclic mitochondrial pathway continually oxidizing acetyl-CoA to CO_2_, spawning NADH and FADH_2_, whose electrons are used in electron transport chain (ETC) to generate ATP for chemical and physical work within the cell [16]. Mitochondrial metabolism plays a role in tumorigenesis [63] and furthermore, major mitochondrial enzymes and pathways reinforce tumor progression induced by key oncogenic drivers [64, 65]. Dominant defects associated with oncogenesis were reported for succinate dehydrogenase (SDH), fumarate hydratase (FH) and isocitrate dehydrogenase (IDH) [66]. These mutations in enzymes underlie the mechanistic rationale on how alterations in the mitochondrial pathway can potentially change bioenergetics of the cell itself. In this chapter we discuss potent TKIs that were shown to perturb pathways and metabolites included in mitochondria metabolism such as TCA components, ETC complexes and metabolites related to oxidative phosphorylation (OXPHOS).

In the already mentioned study focusing on imatinib-treated BCR-ABL-positive cells, the increase of mitochondrial glucose metabolism following treatment by high imatinib concentration (above the IC_50_ value of 2.5 μmol/L) was accompanied by a higher energy state (e.g., with an increase of all phosphate nucleoside triphosphates (NTPs)), being possibly a result of an activation of the TCA cycle together with dysregulation of glucose metabolism [45]. The energy metabolism in BCR-ABL-negative HC-1 cell line was not affected by imatinib [45]. The TCA cycle metabolite α-ketoglutaric acid was significantly reduced upon treatment with the selective MET inhibitor capmatinib in two TNBC mesenchymal-like cell lines MDA-MB-231 and Hs578. Similarly, TCA cycle and central carbon metabolites such as aspartate, fumarate and malate were decreased following erlotinib treatment [30]. Impact on TCA cycle was described in another study using LAD adenocarcinoma cell lines treated with either erlotinib or gefitinib [27]. Despite the unchanged levels of acetyl-CoA following the distribution of these TKIs, other metabolites such as fumarate, malate and citrate were downregulated in EGFRi-responsive HCC827 and PC-9 cells [27]. This suggests that glutaminolysis is decreased after inhibition of EGFR signaling, consistent with the lower expression levels of glutaminase [27]. Moreover, although the inhibition of EGFR signaling downregulated de novo pyrimidine biosynthesis (reported downregulation of phosphorylation of ribosomal protein S6 kinase 1 (S6K), CAD trifunctional multi-domain protein (carbamoyl-phosphate synthetase 2, aspartate transcarbamoylase and dihydroorotase)), adenosine triphosphate levels (ATP) were not affected [27]. It was proposed, that after the treatment with WZ4002, an EGFR inhibitor, ATP levels increased in H1975 cell line. The results were constant with the results for H1993 cell line, exposed to another MET inhibitor, PHA665752, suggesting a reactivation effort of mitochondrial respiration following the treatment with the inhibitors [32]. To support this hypothesis, it has been further shown that ALK inhibition induces upregulation in total ATP levels while downregulating ADP in favour of biomass production (amino acids, lipids) [58]. The evidence from these data points towards the possibility that the reduction in glycolytic flux following ALK inhibition is not a characteristic feature of a viable cell since ATP levels are normally used as a representation of viability [67].

However, similarly to a previous study [27], an enhanced expression of ETC complexes II, III, IV and V was observed using erlotinib for the treatment of EGFR-sensitive HCC827 cells along with increased citrate levels, while no alterations of malate values were detected [32]. Comparable results indicating dysregulation of mitochondria by a TKI were obtained by Guo et al., who reported a deregulation of eight mitochondrial proteins (SLC25A13, NDUFS3, SDHB, UQCRC1, UQCRC2, COX2, COX5A, CYC1) representative of all four components of ETC and a decrease of mitochondrial permeability transition pore (mPTP) in response to the MET inhibitor PHA665752 in gastric carcinoma cell line SNU5 [55]. In a more recent study, Tesori and colleagues described a dose-dependent increase of reactive oxygen species (ROS), 12 h after exposure of the rat hepatocholangiocarcinoma cell line LCSC-2 to sorafenib [60]. Since mitochondria are a major source of ROS, they indicated that the observed increase of ROS is reflecting an impact of sorafenib on these energy sources [60]. Indeed, sorafenib was shown to depolarize mitochondria, interfering with the mitochondrial function and deregulating one of the mitochondrial enzymes, pyruvate dehydrogenase alpha 1 (PDHA1), which catalyses the production acetyl-CoA [60]. Furthermore, ATP levels were reduced, proposing that LCSC-2 cells strongly depend on mitochondrial functionality and that this drug interacts directly with mitochondria [60]. In addition, a 2017 study by Fumarola et al. using FGFR-amplified cell line H1703 reported, that FGFR1 inhibition by dovitinib or NVP-BGJ398 prevented ATP production and that decreased ATP levels caused activation of AMPK, a master energy sensor activated by elevated AMP:ADP ratio within the cell [62]. Aforementioned evidence uncovered novel mechanisms through which inhibitors act on mitochondrial biomarkers such as TCA cycle, NTPs and acetyl-CoA. Although the reported results are not always consistent across distinct TK systems, most of these studies agree, that upon TKI treatment cancer cells develop efforts to reactivate mitochondria and functionality of mitochondrial respiration as a potential saving mechanism against rapidly lethal effects of targeted therapies.

## Metabolism of amino acids and their products

High demand for protein synthesis in tumors boosts the enormous need for amino acids. The mTOR pathway, a signaling cascade mobilized by many different oncogenes, is a one of the major pathways strongly associated with amino acid metabolism [68]. Tumor cells have a particular interest in amino acids such as serine and glycine, which fuel synthesis of nucleotides, proteins and lipids needed for proliferation [69, 70] and asparagine, which regulates the uptake of amino acids, hence the increased asparagine synthetase having role in a drug resistance [71]. Interestingly, amino acid deregulation plays an important function in immune tolerance in cancer [17]. Since T cells need for their proliferation tryptophan, amino acid depleted in many types of cancers, their response to fight this neoplastic phenotype is limited [72]. Furthermore, some cancers are auxotrophic for arginine, an amino acid playing role in urea, ornithine and citrulline production [17, 73]. Considering the influence that amino acid metabolism has on the reprogramming of neoplastic metabolism, we discuss in this section known effects of TKIs on amino acids and their related metabolites and appropriate enzymes.

In a study published in 2015, where the objective was to comparatively profile the metabolite composition of hepatocellular carcinoma HepG2 cells treated solely with sorafenib or everolimus (formerly RAD001, a mTOR inhibitor), and the combination of these two drugs using a NMR-based metabolomic approach, the group of Ji-Xiang Zhang reported that key metabolites are significantly altered in everolimus-treated cells [74]. Aspartate and glutathione disulfide were not changed in sorafenib-treated cells, however, alanine, arginine and glycine were significantly decreased in everolimus-treated cells. When comparing changes occurring between sorafenib and combination treatment, the combination therapy significantly downregulated molecules such as leucine, alanine, arginine and glycine. Combination-treated cells encountered decrease in arginine and increase in valine, lysine, tyrosine and aspartate as compared to the changes induced by the everolimus therapy, thus proposing that sorafenib and everolimus may, in addition to their individually induced effects on the cells, act on the metabolism of HepG2 cells also synergistically [74]. Further, it has been reported that amino acids proline and aspartate were increased following erlotinib treatment in EGFR-sensitive LAD cells [27]. Supporting these findings, a study looking for potential RTK inhibition biomarkers for TNBC models reported that in the basal-like cell line MDA-MB-231, perturbation of amino acid metabolism (e.g., glycine, alanine, cystine, glycolic acid, valine, leucine, proline and tryptophan) occurs upon erlotinib or capmatinib treatment [30]. Moreover, the authors of this study could further demonstrate that suppression of tryptophan metabolism enhances capmatinib treatment [30]. Other recent work highlights significant changes in glycine, serine and threonine metabolism in response to ALK inhibition as a consequence of deregulation of PKM2 [58], which may regulate de novo serine synthesis via 3-phosphoglycerate [75].

Comparable to the aforementioned, metabolic profiling of gefitinib-sensitive myeloma cancer cells LP-1 revealed upregulation of threonine, histidine, proline, asparagine and tyrosine following EGFR inhibition by gefitinib [34]. Related to gefitinib treatment, it has been reported that the concentration of arginine in breast cancer patients is significantly reduced [76]. The results of this study suggest that depletion of arginine in malignancies, for which arginine is auxotrophic, can be exploited as a potential targeted therapy [77]. At this point it is important to clarify that arginine is a nonessential amino acid in a healthy environment, however it is essential for highly proliferating cells [77]. In the aforementioned report by Gent et al., tryptophan, a major determinant marker of metastasis competence, did not change upon EGFR inhibition with the small molecule inhibitor gefitinib, widening the gap between the in vitro findings and their in vivo translation [78].

To fulfil biosynthetic demands associated with proliferation, tumors increase the import of nutrients including amino acids for their survival. Studies discussed in this section suggest that many amino acids are consistently decreased following the treatment with TKs inhibitors. Since most of these reports have been primarily focused on changes in glucose and mitochondrial metabolism, we are only starting to unravel the extent to which amino acids contribute to tumors’ pathology and if fluctuations in their levels that occur upon administration of TKIs could be plausibly considered as markers of therapy efficacy, or are rather merely passengers of events that take place upon inhibition of the respective oncogenic kinases.

## Lipid metabolism

Although phospholipids, fatty acids and cholesterol represent extensive energetic storage and important building blocks for plasma membrane, impact on lipid metabolism in cancer cells received less attention than changes in glucose or amino acid metabolism. At the same time it has been well established that cancerous tissues are defined also by an increased rate of lipid synthesis [79]. Transcription factor sterol regulatory element-binding protein 1c (SREBP-1c) regulated by mTORC1 promotes tumor progression by increasing de novo lipid synthesis [80], which potentially implicates mTORC2 in the control of lipogenesis. Although lipids are extensively used as cancer biomarkers (e.g., phospholipid levels for breast cancer [81] or apolipoprotein A-I for colorectal cancer [82]), our current knowledge concerning the impact of TKIs on lipid metabolites and pathways is rather limited. The aforementioned study by Gottschalk et al. reported a significant decrease of phosphocholine, a precursor for membrane synthesis, as a consequence of the inhibition of cell proliferation in imatinib-treated BCR-ABL-positive cells [45]. At the same time, no changes were detected is BCR-ABL-negative HC-1 cell line following imatinib treatment [45]. It has been proposed that phosphocholine accumulates in different types of tumors (for example in breast, ovary or colon) as a result of an enhanced choline transport into the cells [83–85] and the high increase of phosphocholine is used as a marker for various cancers with higher proliferation rate. Imatinib-induced drop in phosphocholine reported by Gottschalk was accompanied by an upregulation glycerophosphocholine [45], related to apoptotic processes and membrane degradation [86]. In this respect, a 2015 study by Zheng et al. revealed that low dosage of sorafenib treatment affects glycerophospholipid metabolism in hepatocellular carcinoma cells HepG2 [74]. Interestingly, the treatment with non-tyrosine kinase inhibitors, including inhibitors of PI3K and RAS, mostly lead to downregulation of choline-containing metabolite levels, composed of total choline, phosphocholine and glycerophosphocholine [87–89]. In addition, a study conducted by Lanning et al. reported perturbed lipid metabolism which was present in more than 15% of total hits in a metabolomics study assessing responses of TNBC cancer cell lines to EGFR and MET inhibition. Interestingly, MDA-MB-231 and Hs578T cell lines were sensitive to the knockdown of fatty acid genes upon erlotinib treatment whereas capmatinib (INC280) sensitized MDA-MB-468 cells to knockdown of arachidonic and linoleic acid metabolism rate limiting enzymes, providing an additional motivation for co-targeting the metabolic and kinase pathways in TNBC patients [30].

Taken together, although our current expertise regarding alterations in lipid metabolism upon distribution of distinct TKIs is rather limited, the aforementioned results strongly suggest that TK inhibition often leads to a decrease in levels of fatty acid metabolites such as phosphocholine. Given the central role that lipids are playing in tumor development and tumor progression, further investigations regarding potential clinical relevance of TKI-related modulations in lipid metabolism are needed.

## Conclusions

The introduction of TKIs to the armamentarium for the modulation of growth factor signaling has revolutionized treatment outcome of many cancer patients. Nevertheless, acquisition of drug resistance and reported side effects strongly limit their clinical use. Importantly, molecular mechanisms responsible for these complex processes induced by TKIs are not sufficiently understood yet. Metabolomics, either as a unique approach or in use in combination with other omics technologies, is a highly effective approach not only for biomarker discovery but has also the potential to unravel molecular processes that underlie mechanisms of action of various compounds including TKIs.

Nowadays it is relatively well established that TKIs such as imatinib, erlotinib or gefitinib impose metabolic changes on glycolysis profile of cancer cells expressing their respective targets. Indeed, recent studies show that these compounds decrease glucose uptake, potentially affecting major players of glucose metabolism such as transporters and rate limiting enyzmes, and by still unknown mechanisms contribute to side effects such as reactivation effort of mitochondrial respiration. On the contrary, metabolic effects of TKIs on amino acid and lipid metabolism are much less clear and cannot be generalized yet.

In summary, although the current knowledge on TKIs impact on cellular metabolism is continuously expanding, the detailed molecular mechanisms underlying many of the observations described within this review remain largely unknown and further biological investigations are warranted to understand the metabolic on- and off-target effects related to TKIs treatment.

## Abbreviations

ALK: Anaplastic lymphoma kinase
ATP: Adenosine triphosphate
CML: Chronic myeloid leukemia
ECAR: Extracellular acidification rate
EGF(R): Epidermal growth factor (receptor)
ERBB2: Receptor tyrosine-protein kinase erbB-2 precursor
ETC: Electron transport chain
FGF(R): Fibroblast growth factor (receptor)
GLUT: Glucose transporter
GTP: Guanosine triphosphate
HCC: Hepatocellular carcinoma
HIF: Hypoxia-inducible factor
HK2: Hexokinase 2
HNSCC: Head and neck squamous cell carcinoma
LAD: Lung adenocarcinoma
LDHA: Lactate dehydrogenase A
mTOR: Mammalian target of rapamycin
NADPH: Nicotinamide adenine dinucleotide phosphate
NPC: Nasopharyngeal cancer
NSCLC: Non-small cell lung cancer
PFKFB2: 6-phosphofructo-2-kinase/fructose-2,6-bisphosphatase 2
PI3K: Phosphatidylinositol 3-kinase
PKM2: Pyruvate kinase muscle isozyme 2
PPP: Pentose phosphate pathway
ROS: Reactive oxygen species
RTK: Receptors tyrosine kinase
TCA: Tricarboxylic acid
TIGAR: TP53-inducible glycolysis and apoptosis regulator
TKI: Tyrosine kinase inhibitor
TNBC: Triple-negative breast cancer
VEGF(R): Vascular endothelial growth factor (receptor)
